# Microfluidic Cell Trapping for Single-Cell Analysis

**DOI:** 10.3390/mi10060409

**Published:** 2019-06-19

**Authors:** Bing Deng, Heyi Wang, Zhaoyi Tan, Yi Quan

**Affiliations:** Institute of Nuclear Physics and Chemistry, China Academy of Engineering Physics, Mianyang 621900, China; Collaborative Innovation Center of Radiation Medicine of Jiangsu Higher Education Institutions, Suzhou 215000, China; hywang@caep.ac.cn (H.W.); tanzhaoyi@caep.ac.cn (Z.T.)

**Keywords:** cell trapping, microfluidic, microcavity, U-shaped, flow short cut

## Abstract

The single-cell capture microfluidic chip has many advantages, including low cost, high throughput, easy manufacturing, integration, non-toxicity and good stability. Because of these characteristics, the cell capture microfluidic chip is increasingly becoming an important carrier on the study of life science and pharmaceutical analysis. Important promises of single-cell analysis are the paring, fusion, disruption and analysis of intracellular components for capturing a single cell. The capture, which is based on the fluid dynamics method in the field of micro fluidic chips is an important way to achieve and realize the operations mentioned above. The aim of this study was to compare the ability of three fluid dynamics-based microfluidic chip structures to capture cells. The effects of cell growth and distribution after being captured by different structural chips and the subsequent observation and analysis of single cells on the chip were compared. It can be seen from the experimental results that the microfluidic chip structure most suitable for single-cell capture is a U-shaped structure. It enables single-cell capture as well as long-term continuous culture and the single-cell observation of captured cells. Compared to the U-shaped structure, the cells captured by the microcavity structure easily overlapped during the culture process and affected the subsequent analysis of single cells. The flow shortcut structure can also be used to capture and observe single cells, however, the shearing force of the fluid caused by the chip structure is likely to cause deformation of the cultured cells. By comparing the cell capture efficiency of the three chips, the reagent loss during the culture process and the cell growth state of the captured cells, we are provided with a theoretical support for the design of a single-cell capture microfluidic chip and a reference for the study of single-cell capture in the future.

## 1. Introduction

Mammalian eukaryotic cells generally are between 1–10 μm in diameter, and the content of a single cell is at the fL level [1]. The culture micro-environmental scale, composed of the extracellular secretory protein and the attachment matrix, is in the range of several tens to several hundreds of micrometers. Therefore, if researchers hope to obtain new discoveries at the single cell or even sub-cell level around intercellular interactions, cellular and external environmental effects and intracellular signaling pathways, an analytical tool that matches the research object in dimensions is indispensable. With the deepening of the exploration of the laws of life, the demand for real-time and dynamic research methods has led to the emergence of new life analysis techniques and methods [2,3,4,5,6,7,8]. Lab-on-a-chip (LOC) was considered as a breakthrough technology, on the basis of its good manipulation of small volume liquids, such as cell isolation, localization and capture, to enable diverse cell-related studies at cellular, subcellular and molecular levels that can be performed at the micron scale. Some examples are fluid mixing devices on microfluidic chips [9,10], concentration gradient generating devices [11], the screening and separation of different types of cells [12] and even building tissue and organ models on a chip [13]. The applications of the chip have proven its importance in fundamental biology studies and clinical diagnosis [14,15,16,17]. These applications and advantages make LOC an important technical support in the field of cell life science research. Moreover, cell analysis based on microfluidic chips has also facilitated the application of this technology in life sciences and related research fields. The combination of microelectronic technology and other physical/chemical units provide high-throughput single-cell analysis, and are able to obtain variety of bio-information. In addition, the transparency of a chip makes the optical observations of the dynamic process of a cell possible, especially as the chip can provide a living environment closer to the cells in the living body for cell research in vitro [18,19]. In particular, the applications of LOC in cell biology-related research have shown important practical significance for revealing disease mechanisms [20,21,22], screening drug targets [23,24,25] and developing new drugs [26,27,28].

As one of the important biotechnologies, LOC not only has a wide range of application prospects in multicellular culture and analysis, but also has been established as an enabling technology in single-cell studies [29,30,31,32,33,34]. Conventional biological studies in the past have generally regarded cell samples as homogeneous and stable, with an average cell population response to cell proliferation, differentiation and cellular responses to external stimuli. In reality, however, cells are usually heterogeneous [19]. With the advent and development of LOC, this technology provides the possibility to accurately manipulate the flow of the medium in the order of micrometers or nanometers [35], which brings an opportunity to overcome the shortcomings of the traditional in vitro cultured cell method, enabling the intrinsic information of individual cells to decouple noise from population heterogeneity and enabling research on protein localization and kinetics. Recent reports show that droplet microfluidics is among the most promising candidates for capturing and processing thousands of individual cells for whole-transcriptome or genomic analysis in a massively parallel manner with minimal reagent use [36]. In addition, microfluidic chip technology can manipulate the number and density of cells within a defined volume and can detect the morphology or physiological environment of a single cell, which is an accuracy that cannot be achieved by conventional culture devices. Recent literature reports that using a modified microfluidic cell-culture device with an image analysis pipeline for robust lineage reconstruction allowed simultaneous tracking of many cells over multiple generations, and revealed that cells expand exponentially throughout their cell cycle [37]. Lydia Robert et al. [38]—by combining microfluidics, time-lapse imaging and a fluorescent tag of the mismatch repair system in *Escherichia coli*—visualized the emergence of mutations in single cells and enabled the investigation of single-cell individuality in mutation rate, mutation fitness costs and mutation interactions.

The premise of single-cell level research is to achieve single-cell capture, and then to achieve subsequent experiments, such as single cell pairing, fusion, lysis and intracellular composition analysis. As far as we know, we can separate the microfluidic single cell trapping into three categories according to the contacting of a cell to the surface during the capture process: (1) Surface contact capture—mainly including capture by chemical method and capture by hydrodynamic method [39,40,41]. (2) Non-surface contact capture—mainly including capture using electrophoresis [42,43,44], magnetic force [45], acoustic force [46,47,48], sound wave, etc. [49]. (3) Capture between surface contact and non-surface contact—mainly by means of colloids (e.g., hydrogels) [22,23,24]. The single-cell capture method based on fluid dynamics can construct fluidized channels to fix cells in different sizes for specific cells of different sizes. The realization of single-cell capture is the basis of cell culture, cell fusion, cell pairing and other operations, while a hydrodynamic microfluidic device can accurately control fluid flow, reduce cell culture time and cost, can provide cell analysis with the advantages of minimum dilution error, can achieve a sufficient number of individual cells to analyze the cell, can reveal the characteristics of individual cells and the differences between cells and can obtain more accurate and statistically significant data. As a result, single-cell capture based on fluid mechanics has received more and more attention [50].

Based on this, we made three different types of microfluidic structures for single cell capture, successfully demonstrated the cell capture ability of different structural chips and compared the controllability and feasibility of cell growth and single-cell observation. The research results provide reference for different cell culture needs, and provide a reference for cell-level research, such as organ chip construction, analysis of single-cell stress response, interaction between cells and cells and also for the development of radioactive cells. This provides a reference for a more efficient and environmentally friendly research platform for the biological effects of experimental research.

## 2. Experimental Setup

### 2.1. Materials and Methods

#### 2.1.1. Cell Culture and Preparation

All experiments were performed with human bone marrow derivate mesenchymal stem cells (hMSCs), and cells were incubated at 37 °C with 5% CO_2_ in basal medium supplied with 7% fetal bovine serum, 15 ng/mL rhIGF-1 (recombinant human IGF-1), 125 pg/mL RhFGF-b (Recombinant humanFGF–Basic), 2.4 mmol/L L-alanyl-L-glutamine (ATCC PCS-500-041). hMSC cells were purchased from the Shanghai Cell Bank of the Chinese Academy of Sciences. Before seeding the cell on the chip, adherent cells were digested with Accutase digestible solution (Invitrogen, Thermo Fisher, Waltham, MA, USA), then cells were counted and resuspended in medium to obtain a cell population of 1 × 10^6^ cells/mL. 

#### 2.1.2. Cell Seeding

Before the cell suspension was injected into the chip, the chip was washed with medium and stayed overnight in the incubator. As mentioned above, cell suspension with a cell population of 1 × 10^6^ cells/mL was infused into the microfluidic culture chambers by a sterile syringe. The infusion rate was controlled using a programmable syringe pump (Pump 11, Harvard Apparatus, Holliston, MA, USA) at a flow rate of 5–50 μL/min through the chip for 5–20 min. Before a desired loading density appeared in the culture chamber, the loading rate slowed down to 5 μL/min to keep the fluid flowing and avoid causing a cell blockage in the channel. The microarray after cell seeding was placed in a Petri dish and cultured in an incubator.

#### 2.1.3. Chip Fabrications

In the experiment, the PDMS (polydimethylsiloxane) chip was molded from a SU-8 (photoresist) template, which was fabricated by photolithography using a slide as substrate. The slides were first ultrasonically cleaned with acetone, ethanol and pure water separately. Then, they were baked at a high temperature and treated in O_2_ plasma to remove moisture from the substrate surface. After preparing the substrate, SU-8 photoresist (GM 1060, Gersteltec, Pully, Switzerland) was spin-coated on it at a low speed of 300 rpm for 70 s and a high speed of 1750 rpm for 40 s. Next, the resist was baked at 60 °C for 10 min and 95 °C for 2 h as a gradient heating to remove the solvent in resist, which is called pre-bake. The substrate without solvent was then exposed to UV (ultraviolet) light at a wavelength of 365 nm for 21 s with an optical power of 24 mW/cm^2^ under a photomask. Thereafter, post-baking was performed on the substrate using heating at 65 °C for 2 min and at 95 °C for 1 min to cure the resist. In the developing process, the developer propylene glycol methyl ether acetate (PGMEA) was sprayed onto the substrate at a flow rate of 400 mL/min within 2 min to wash away redundant resist, and then rinsed with isopropyl alcohol to dissolve the developer. After developing, the surface was gently dried with a high pressure air gun. The developed substrate was finally baked at 150 °C for 30 min to harden the resist film. Before molding the substrate by PDMS, the surface of the substrate was hydrophobized by 1H,1H,2H,2H-perfluorooctyl trichlorosilane, and baked at 150 °C for 10 min to cure the hydrophobic layer. Then, SYLGARD 184 (Dow Corning, Midland, MI, USA) silicone rubber was casted onto the substrate at a 10:1 ratio of part A and B and placed in an oven at 60 °C for 4 h. Finally, the cured PDMS was transferred from the substrate and was demolded, cut, punched, cleaned and bonded to clean slides.

## 3. Results

### 3.1. Microcavity Cell Trapping

**Figure 1:**
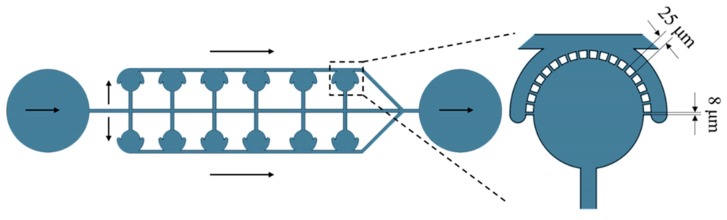
Schematic diagram of microcavity cell trapping structure.

The microcavity structure can be used for the capture of a small number of cells, allowing a small number of cells to grow together with the same microenvironment without affecting cell–cell interactions and analyzing individual cells. The schematic diagram of the microcavity structure is shown in Figure 1. The microcavity structure was a row of the same size circular chambers of 175 μm radius. The liquid flowed into the chamber from the main channel (50 μm wide) and flowed out into the upper channel into the top through an array of micropillar blocking structures, having a pitch of 8 μm and a width of 25 μm. The outflow position was arranged with a blocking structure to prevent cells from flowing out with the medium.

**Figure 2:**
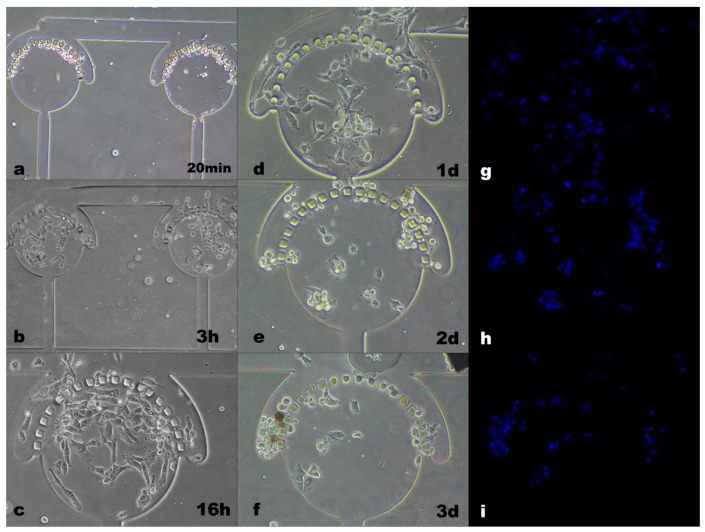
Distribution and growth state of cells cultured continuously in microcavities.

The distribution of hMSC cells in the microcavity structure and the culture results of the cells in the microcavity within 16 h can be seen in Figure 2. As shown in Figure 2a, cells were suspended and concentrated in the top of the microcavity at 20 min after they passed into the tube. After 3 h incubation, most of the cells returned to the center of the microcavity with negative pressure and adherent to the glass substrate to grow, while a small amount of cells escaped through the barrier to the outside of the cavity. At 16 h post-cell injection, cells had anchorage-dependent growth in the microcavity, and the confluence reached 80% to 90%. Combined with the observation of cell morphology, this showed that these cells grew well, as it had efficient proliferation. Figure 2d is a photograph of cells after continuous culture for 1 d, at which time the cell has undergone apoptosis (combined with fluorescence pictures Figure 2g–i, the changes in cell nucleus exhibiting rounding, shrinkage, blebbing of the plasma membrane, condensation and posterior fragmentation of the chromatin, which are the characteristics of apoptotic cells). After continuous culture for 2 days and 3 days (shown in Figure 2e,f) the apoptotic cells shed into a mass and entered the cavity through the gap in the barrier.

It can be seen from Figure 2 that the structure of the microcavity chip was favorable for cell capture and culture, and the cell flux was large. At the same time, however, the number of cells entering the microcavity was too large. Although most cells adhered to the spindle type within 16 h and the growth state was good, the cell superposition growth phenomenon was serious, which was not conducive to further observation and analysis of single cells. Moreover, if the medium cannot be changed in time in the microcavity microenvironment, the sufficient nutrients of multiple cells cannot be ensured and metabolic wastes are taken away, thereby causing apoptosis of the cells. However, frequent fluid changes can destroy the continuous reaction of stem cells in and around the microcavity, and thus cannot maintain their differentiated or undifferentiated or proliferated state. 

### 3.2. U-Shape Capture

**Figure 3:**
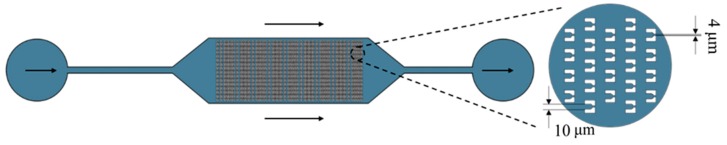
Schematic diagram of the U-shaped cell trapping structure.

**Figure 4:**
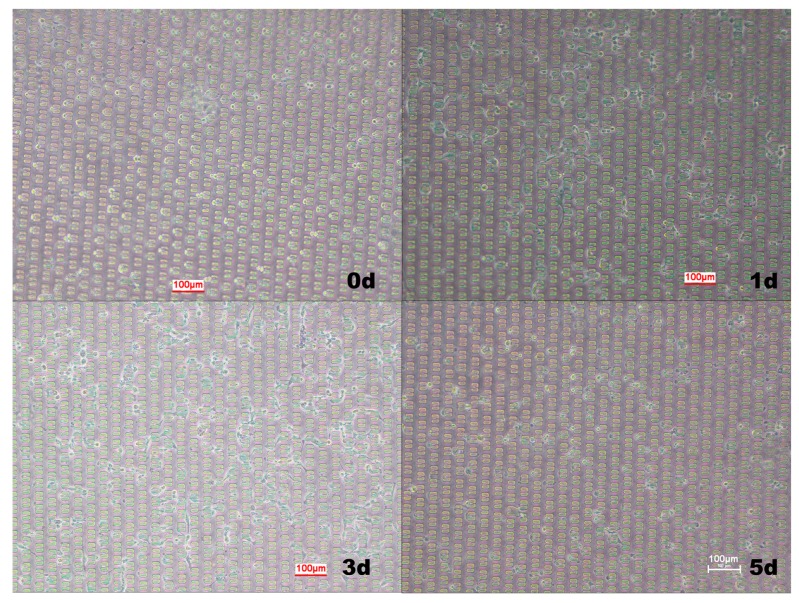
Distribution and growth state of cells cultured continuously in the U-shape capture.

The U-shaped structure is formed by splicing two small bodies that are entangled together [51]. The schematic diagram of the U-shape capture structure is shown in Figure 3. When the fluid passed through the U-shaped groove, the cells entered the larger opening (10 μm wide) and were trapped, while the smaller opening (4 μm wide) released the solution. All the channel heights were 25 μm. The U-shaped structure can achieve single cell capture. Figure 4 shows the capture and distribution of hMSC cells by U-shaped structure chips and the culture results of cells in the chip within 5 days. It can be seen that the U-shaped structure had high capture efficiency for single cells, was evenly distributed in the chip and was filled with medium around the cells. At 24 h after cell capture, 90% of the cells adhered well and the number of cells did not change significantly. After 2 days of culture, the adherent and well-growth cells split in the chip, which increased the number of cells in the chip, indicating that the cells gradually adapted to the growing environment in the chip. After 5 days, the cells in the chip showed apoptosis. The decrease in the number of cells was caused by the increase of cellular metabolic waste and the decrease of nutrients caused by long-term culture, which caused the morphology of the cells to change from the adherent shuttle type to the agglomerated sphere, and the gradual emergence of contact growth inhibition caused apoptosis. The U-shaped structure allowed stem cells to grow and proliferate in the chip under conditions of infrequent medium exchange, which is beneficial to reduce the use of valuable reagents or toxic reagents. At the same time, because the spacing between the U-shaped structures was far enough, cell adherent growth did not cause cell overlap, and thus subsequent observation and analysis of single cells could be achieved; however, the disadvantage is that the cells, after adherence, were likely to cause a blockage of the pores between the U-shaped structures.

### 3.3. Flow Shortcut Structure

**Figure 5:**
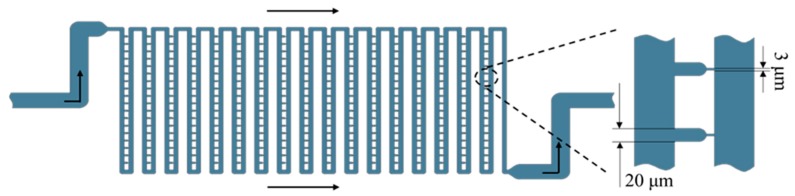
Schematic diagram of the flow shortcut structure.

**Figure 6:**
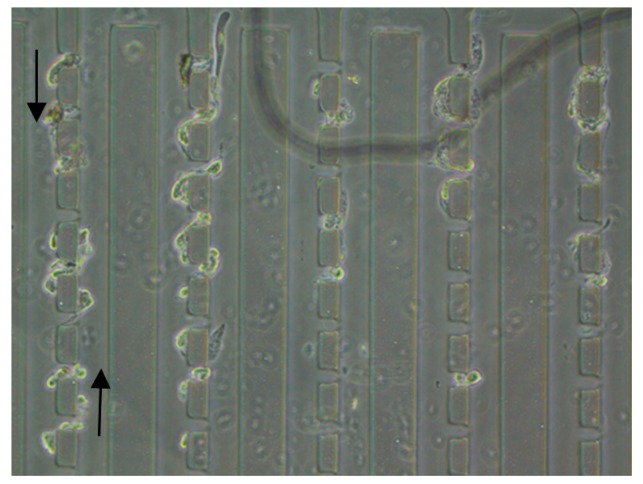
Cell distribution and growth state after flow shortcut structure capture for 3 days.

The flow shortcut structure achieved single cell capture by fluid flowing through the gaps of the cell size calibrated on the chip array (Fluidigm Corporation, South San Francisco, CA USA). Figure 5 shows the schematic of this structure. The main channel width of this structure was 50 μm, the gap for capturing cells was 20 μm and the narrow gap at the back was 3 μm for releasing the solution. The channel height was 23 μm. Since the channel for liquid flow was specifically designed, the path of the fluid flow was not blocked by the adherent growth of the cells; the cells were evenly distributed and distributed in an array. However, because of the higher fluid flow efficiency, the single cell capture efficiency of the flow shortcut structure was lower than that of the U-type structure, and the sample waste was severe. Figure 6 shows the growth and distribution of the captured cells after 3 days of incubation in the chip. It can be seen that the captured cells adhered well to the growth state, and the flow shortcut structure could realize the single cell capture and subsequent observation and analysis of the captured single cells; however, attention should be paid to the rate at which the cell sample was infused into the chip to prevent severe cell deformation due to shear forces of the fluid, thereby interfering with the normal physiological function of the cell.

## 4. Discussion and Conclusion

The effects of different chip structures on the captured cells were not only on the distribution of the captured cells in the chip, but also on the concentration of nutrients and metabolites around the cells, thereby affecting the growth and apoptosis time of the cells in the chip. At the same time, the state of cell-adherent growth could further affect the observation and analysis of single cells. The microcavity structure had a large chip flux and less waste on the sample, and thus a small amount of cells could be co-cultured in the same micro environment, and could be used for cell cluster analysis or tissue and organ simulation (pulmonary chip) [52,53,54,55,56], as well as the simulation of complex structures, microenvironments and physiological functions of human organs. However, cells will overlap heavily after adherent growth in the microcavity structure, and the cell position is not fixed, making it is difficult to observe single cells. In addition, cells cultured continuously in the microcavity structure showed apoptosis on the first day, and cells were continuously cultured in the chip for a significantly shorter time than the other two structures. Moreover, the microcavity structure is prone to collapse during the chip fabrication process, and the rigidity of the fabricated material is required. Thus, the culture microenvironment provided by the single cell capture structure is more conducive to cell growth than the co-culture of a small number of cells provided by the microcavity structure. The U-shaped structure had a high density of chip arrays, enabling single cell capture and long-term continuous culture of the captured cells, and apoptosis began to occur after 5 days of continuous cell culture. However, the U-shaped structure had the disadvantage that the cells trapped in the chip were unevenly distributed and were liable to be clogged at the edge of the chip. The flow shortcut structure enabled single-cell capture and cell-adherent growth, and it was easy to observe and analyze single cells. However, the shortcut structure used to capture cells had a low structural density, resulting in low cell-capture rates and large sample wastage. According to our experiments, the U-type structure is more efficient in capturing single cells with less sample and reagent consumption than the flow shortcut structure. Although the microcavity structure had high cell capture efficiency and flux, the cells in the microcavity structure tended to overlap after adherence, which was not conducive to the observation and analysis of single cells.
